# Multiple Opioid Prescribers During the Perioperative Period Increases Opioid Consumption Following Upper Extremity Surgery: A Multicenter Analysis

**DOI:** 10.7759/cureus.24541

**Published:** 2022-04-27

**Authors:** Joseph Paladino, Clay B Townsend, Justin Ly, Ryan Judy, Christine Conroy, Shivangi Bhatt, Hesham Abdelfattah, Mark Solarz, Katharine Woozley, Asif M Ilyas

**Affiliations:** 1 Department of Orthopaedics, Sidney Kimmel Medical College, Thomas Jefferson University, Philadelphia, USA; 2 Orthopaedic Surgery, Rothman Orthopaedic Institute, Philadelphia, USA; 3 Orthopaedics, Lewis Katz School of Medicine, Temple University, Philadelphia, USA; 4 Department of Orthopaedics, Einstein Healthcare Network, Philadelphia, USA; 5 Orthopaedic Surgery, Drexel University College of Medicine, Philadelphia, USA; 6 Orthopaedic Surgery and Sports Medicine, Temple University Hospital, Philadelphia, USA; 7 Department of Orthopaedics, Rothman Orthopaedic Institute, Thomas Jefferson University, Philadelphia, USA; 8 President, Rothman Orthopaedic Institute Foundation for Opioid Research & Education, Philadelphia, USA

**Keywords:** orthopedic surgery shoulder and elbow and upper extremity, multiple prescribers, prescription drug monitoring program, perioperative pain management, opioid medication

## Abstract

Background

Opioid prescribing practices have been an area of interest for orthopedic surgeons in the wake of the opioid epidemic. Previous studies have investigated the effects of a multitude of patient-specific risk factors on prolonged opioid use postoperatively. However, to date, there is a lack of studies examining the effects of multiple prescribers during the perioperative period and their potential contribution to prolonged opioid use postoperatively. This study aimed to investigate if multiple unique opioid prescribers perioperatively predispose patients to prolonged opioid use following upper extremity surgery. Second, we compared opioid prescribing patterns among different medical specialties.

Methodology

This retrospective study was conducted at three academic institutions. Between April 30, 2018, and August 30, 2019, 634 consecutive patients who underwent one of three upper extremity procedures  were included in the analysis: carpal tunnel release (CTR), basal joint arthroplasty (BJA), or distal radius fracture open reduction and  internal fixation (DRF ORIF). Prescription information was collected using the state Prescription Drug Monitoring Program (PDMP) online database  from a period of three months preoperatively to six months postoperatively. A Google search was performed to group prescriptions by medical specialty.  Dependent outcomes included whether patients filled an additional opioid prescription postoperatively and prolonged opioid use (defined as opioid use three to six months postoperatively).

Results

In total, 634 patients were identified, including 276 CTRs, 217 DRF ORIFs, and 141 BJAs. This consisted of 196 males (30.9%) and 438 females (69.1%) with an average age of 59.4 years (SD: 14.7 years). By six months postoperatively, 191 (30.1%) patients filled an additional opioid prescription, and 89 (14.0%) experienced prolonged opioid use. In total, 235 (37.1%) patients had more than one unique opioid prescriber during the study period (average 2.5 prescribers). Patients with more than one unique opioid prescriber were significantly more likely to have received overlapping opioid prescriptions (15.7% vs. 0.8%, p<.001), to have filled an additional opioid prescription postoperatively (63.8% vs 10.3%, p<.001), and to have experienced prolonged opioid use postoperatively (35.3% vs 1.5%, p<.001) compared to patients with only one opioid prescriber. Patients with multiple unique prescribers filled more opioid prescriptions compared to those with a single prescriber (2.8 refills vs 1.8 refills, p=.035). Within six months postoperatively, 71.4% of opioid refills were written by non-orthopedic providers. Opioid refills written by non-orthopedic prescribers were written for a significantly greater number of pills (68.4 vs. 27.9, p<.001), for a longer duration (22.2 vs. 6.2 days, p<.001), and for larger total morphine milligram equivalents per prescription (831.4 vs. 169.8, p<.001) compared to those written by orthopedic prescribers.

Conclusions

Patients with multiple unique opioid prescribers during the perioperative period are at a higher risk for prolonged opioid use postoperatively. Non-orthopedic providers were the highest prescribers of opioids postoperatively, and they prescribed significantly larger and longer prescriptions. Our findings highlight the value of utilizing PDMP databases to help curtail opioid overprescription and potential adverse opioid-related outcomes following upper extremity surgery.

## Introduction

Opioid prescribing practices remain a focus for orthopedic surgeons during the current opioid epidemic. Numerous strategies, such as multimodal pain regimens and prescribing protocols, have emerged to guide prescribers and help mitigate the adverse outcomes related to prescription opioid addiction [1-5]. Nonetheless, the United States remains the top prescriber of opioids globally [6]. The adverse effects of increased opioid prescription and consumption are far-reaching. Opioid misuse is a proven precursor to addiction, and in 2016 alone nearly 12 million Americans over the age of 12 reported opioid misuse [7]. Opioid addiction imposes a considerable financial burden on patients and the broader healthcare system, in addition to its contributions to patient morbidity and mortality [8]. Specifically, prescription opioids are estimated to have contributed to 50.9% of opioid-related deaths in 2016, with a financial impact in the tens of billions of dollars on the healthcare system [9-11].

Pain management protocols following hand surgery can vary widely among surgeons. However, a shared focus for surgeons is to identify patients at risk for prolonged opioid use postoperatively.  Numerous studies have linked preoperative opioid use to chronic postoperative opioid dependency [12-14]. A potential contributing factor to the widespread availability of opioids is multiple providers prescribing opioids to a single patient. Previous studies have indicated that orthopedic surgeons represent only a small subset of the total opioid prescribers for perioperative patients [15]. One study reported that for patients treated for joint osteoarthritis, 92% of opioid prescriptions were written by medical providers outside of orthopedics, with internal and family medicine accounting for 73.1% of all prescriptions [16]. Additionally, non-orthopedists were found to prescribe a greater number of prescriptions, dosages, and refills per patient compared to their orthopedic counterparts [16]. The lasting effects of postoperative patients receiving opioid prescriptions from multiple unique prescribers warrant closer investigation [17].

Many studies have investigated risk factors for prolonged opioid use following orthopedic surgery; however, very few have focused on the effects of multiple unique opioid prescribers on prolonged postoperative opioid use. The purpose of the current study was to investigate if having multiple individual opioid prescribers in the perioperative period predisposes patients to prolonged opioid use following hand and upper extremity surgery. Second, we sought to examine from which medical specialties patients receive the majority of opioid refills postoperatively. We hypothesized that patients with multiple unique opioid prescribers would fill more prescriptions and that orthopedic providers would represent the prescribing minority during the postoperative period.

## Materials and methods

Inclusion and exclusion criteria

Following multicenter Institutional Review Board approval, including a waiver of informed consent (Thomas Jefferson University, #20E.1221), this retrospective study was conducted at three urban academic institutions. An electronic medical record database query was performed at each institution to identify all patients ≥18 years old who from April 30, 2018, to August 30, 2019, underwent one of three isolated procedures: carpal tunnel release (CTR, CPT 64721), basal joint arthroplasty (BJA, CPT 25447), or distal radius fracture open reduction and internal fixation (DRF ORIF, CPT 25609). Surgeries were performed by one of 14 board-certified and fellowship-trained orthopedic hand and upper extremity surgeons. All patients in this study only had a single surgery during the study period.

Data collection

Patient prescription and prescriber information was manually collected using the Pennsylvania Prescription Drug Monitoring Program (PDMP) website. The Pennsylvania PDMP system contains prescription data from 19 states, including Pennsylvania, Arkansas, Connecticut, Delaware, Florida, Louisiana, Maine, Maryland, Massachusetts, Military Health System, Minnesota, New York, North Carolina, Ohio, Oklahoma, Rhode Island, South Carolina, Texas, Virginia, and West Virginia. Prescriptions were analyzed from a period of three months preoperatively to six months postoperatively. The types of opioids prescribed, prescription strength, written/filled dates, pill numbers, duration, and morphine milligram equivalents (MMEs) per prescription were among the data variables collected. The number of unique opioid prescribers during the study period were tabulated for each patient. A Google search was then performed to identify the medical specialty of each individual opioid prescriber to compare prescribing patterns between orthopedic and non-orthopedic providers. Dependent outcomes included (1) filling an additional opioid prescription postoperatively, and (2) prolonged opioid use between three and six months postoperatively.

Statistical analysis

Categorical data are presented as count (percentage) and analyzed with the chi-square test. Continuous data are presented as mean (standard deviation). Continuous data were tested for normality using the Shapiro-Wilk test. Normally distributed continuous data were analyzed with the t-test, and non-normally distributed continuous data were analyzed using the Mann-Whitney U test. Statistical significance was set at p < 0.05.

## Results

A total of 634 patients met the inclusion criteria. By procedure type, this included 276 CTRs, 217 DRF ORIFs, and 141 BJAs. There were 196 males (30.9%) and 438 females (69.1%) with a mean age of 59.4 years (SD = 14.7 years). Evidence of preoperative opioid use was observed in 28.5% (181/634) of patients. Within six months postoperatively, 30.1% (191/634) of patients filled additional opioid prescriptions. Prolonged opioid use three to six months postoperatively was observed in 14.0% (89/634) of patients. There was no difference in the rates of prolonged opioid use between the three surgery types included in this study (p > 0.05).

The 30.1% of patients who filled an additional opioid prescription postoperatively filled a mean of 2.6 (SD = 2.7) additional prescriptions. Over one-third of patients in this study (37.1%, 235/634) received opioid prescriptions from more than one unique medical provider during the study period. These patients filled opioid prescriptions from an average of 2.5 (SD = 0.98, range = 2-9) unique opioid prescribers during the study period.

**Figure 1:**
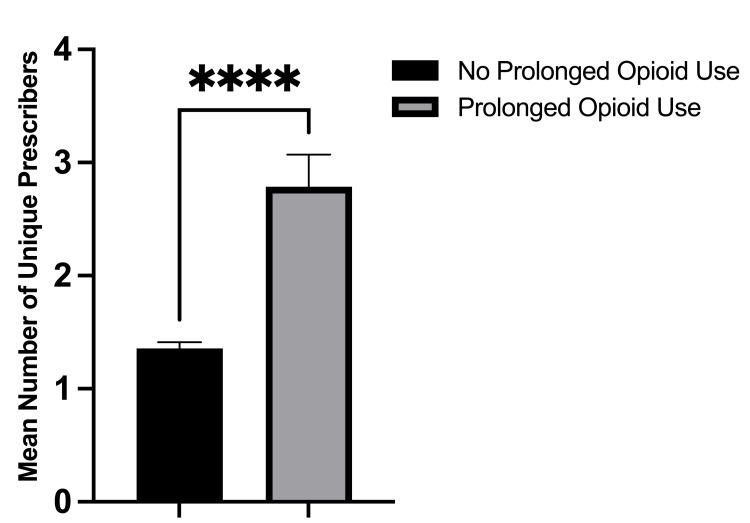
Mean number of unique opioid prescribers for patients with prolonged postoperative opioid use versus patients without prolonged use.

**Figure 2:**
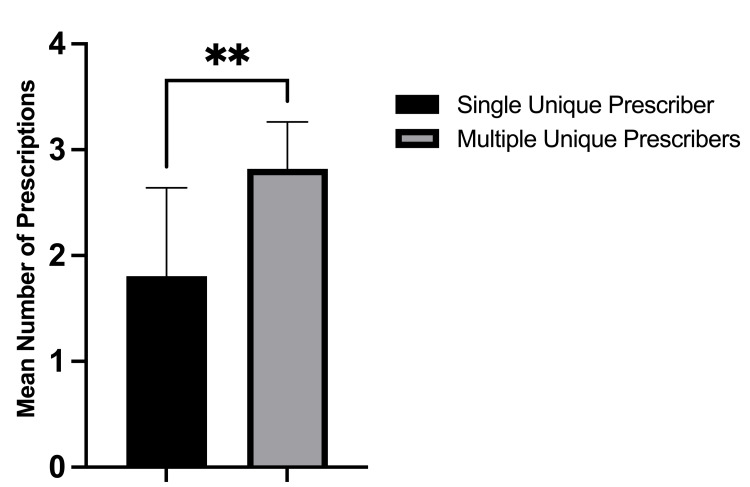
Mean number of opioid prescriptions filled by patients who had a single unique prescriber versus patients with multiple unique prescribers.

There was no difference in age or gender between those that only had a single prescriber versus those with multiple prescribers (Table 1). Patients with more than one opioid prescriber were significantly more likely to have received overlapping opioid prescriptions (15.7% vs. 0.8%, p < 0.001) compared to patients with a single opioid prescriber. This finding is noteworthy because the Centers for Disease Control and Prevention (CDC) lists receiving overlapping opioid prescriptions as a risk factor for developing opioid dependence [18]. Patients with more than one unique opioid prescriber were significantly more likely to fill an additional opioid prescription postoperatively compared to patients with a single opioid prescriber (63.8% vs. 10.3%, p < 0.001). Moreover, patients with multiple prescribers were significantly more likely to experience prolonged opioid use postoperatively (35.3% vs. 1.5%, p < 0.001). Patients with prolonged postoperative opioid use were found to have significantly more unique opioid prescribers compared to patients without prolonged opioid use (mean 2.8 vs. 1.4 prescribers, p < 0.001, Figure 1). Of the 191 patients who filled additional opioid prescriptions postoperatively, those with multiple unique prescribers filled significantly more opioid prescriptions than those with a single unique prescriber (2.8 refills vs. 1.8 refills; p = 0.035, Figure 2).

**Table 1:** Demographics and outcomes between patients who only had one unique opioid prescriber versus those with more than one unique opioid prescriber.

	One unique opioid prescriber (N = 399)	More than one unique opioid prescriber (N = 235)	P-value
Age	59.7 (14.9)	58.9 (14.2)	0.54
Gender			0.95
Female	276 (69.2%)	162 (68.9%)	
Male	123 (30.8%)	73 (31.1%)	
Overlapping opioid prescriptions			<0.001
No	396 (99.2%)	198 (84.3%)	
Yes	3 (0.8%)	37 (15.7%)	
1+ opioid refill postoperatively			<0.001
No	358 (89.7%)	85 (36.2%)	
Yes	41 (10.3%)	150 (63.8%)	
Prolonged 3–6-month opioid use			<0.001
No	393 (98.5%)	152 (64.7%)	
Yes	6 (1.5%)	83 (35.3%)	

**Figure 3:**
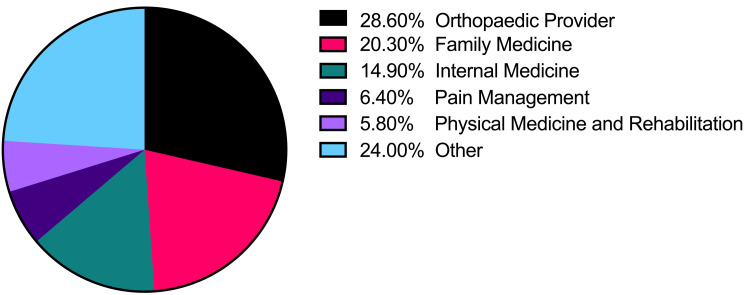
Percentage of postoperative (within six months) opioid prescriptions written by medical specialty.

**Figure 4:**
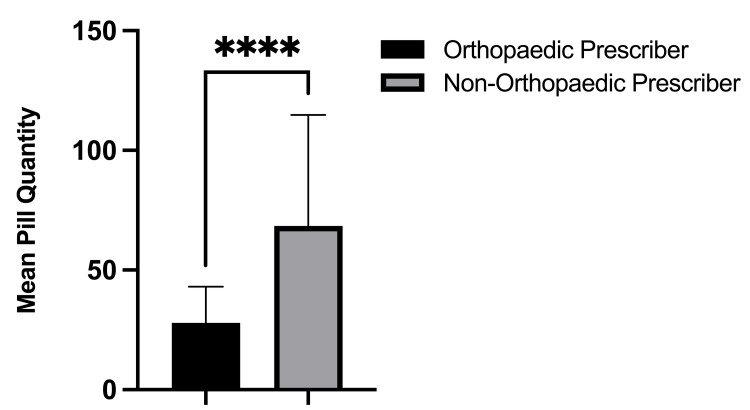
Mean number of pills prescribed per opioid prescription written by orthopedic versus non-orthopedic prescribers during the postoperative period.

**Figure 5:**
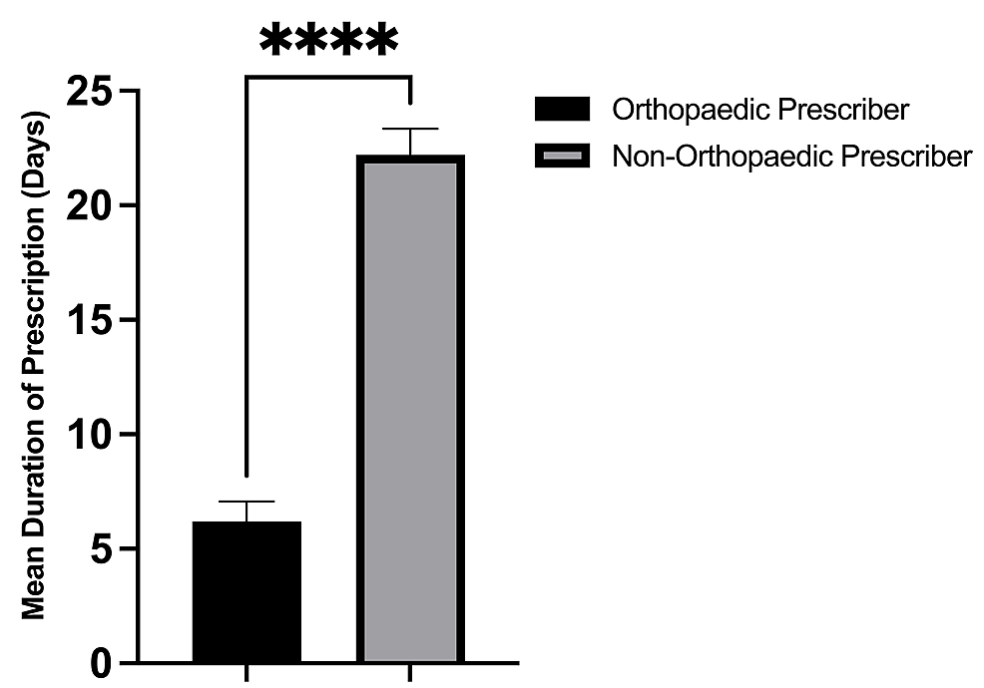
Mean duration of opioid prescription in days written by orthopedic versus non-orthopedic prescribers during the postoperative period.

**Figure 6:**
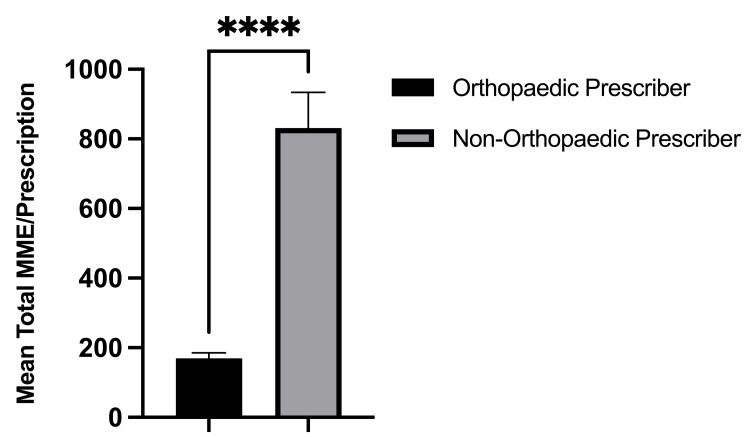
Mean total MMEs/prescriptions written by orthopedic versus non-orthopedic prescribers during the postoperative period. MME: morphine milligram equivalent

On the day of the surgery, 100% (634/634) of opioid prescriptions were written by an orthopedic provider, either the operating surgeon or a mid-level provider. Within the six-month postoperative period, only 28.6% (142/497) of all opioid prescriptions filled by the study patients were written by an orthopedic provider. Alternatively, 71.4% of all postoperative opioid refills within six months postoperatively were written by non-orthopedic providers. The most common non-orthopedic prescriber specialties included family medicine (20.3%), internal medicine (14.9%), pain management (6.4%), and physical medicine and rehabilitation (5.8%) (Figure 3). During the six-month postoperative period, the opioid prescriptions written by non-orthopedic prescribers were written for a significantly greater number of pills (68.4 vs. 27.9; p < 0.001, Figure 4), were written for a significantly longer duration in days (22.2 vs. 6.2; p < 0.001, Figure 5), and were significantly larger in total MMEs (831.4 vs. 169.8; p < 0.001, Figure 6) than prescriptions written by orthopedic prescribers (Table 2).

 

**Table 2:** Postoperative opioid prescription characteristics prescribed by orthopedic versus non-orthopedic providers within six months postoperatively. MME: morphine milligram equivalent; SD: standard deviation

	Orthopedic prescriber	Non-orthopedic prescriber	P-value
Number of prescriptions written	142 (28.6%)	355 (71.4%)	
Quantity of pills	27.9 (SD = 15.1)	68.4 (SD = 46.4)	<0.001
Length of prescription (days)	6.2 (5.2)	22.2 (10.9)	<0.001
Total MME/Prescription	169.8 (94.4)	831.4 (980.1)	<0.001

## Discussion

Despite increased national attention and dedicated public health efforts, the opioid epidemic continues to have devastating impacts on the American populace and the national healthcare system. A recent CDC report revealed that in 2018, 47,000 Americans died from an opioid-related overdose, and an additional two million Americans met the criteria for opioid use disorder [19]. During the same period, the total cost of treating patients with opioid use disorder together with the cost of opioid overdose deaths totaled over $1,021 billion [20]. More recent data indicate that the opioid epidemic shows no signs of slowing. During a 12-month period ending in April 2021, over 75,000 people died from an opioid overdose, which had increased from 56,000 in the previous 12-month period ending in April 2020 [21]. Patients undergoing surgery may be prescribed opioids for pain control postoperatively, placing surgical patients at risk for the detrimental effects of opioid misuse and addiction. A recent study revealed that between 6% and 10 of opioid-naive patients undergoing minor or major surgeries would go on to persistently use opioids postoperatively [22]. These troublesome findings have led to an increased focus on opioid prescribing practices, especially by surgeons looking to prevent patients from experiencing poor postoperative outcomes.

This study analyzed the effects of orthopedic hand and upper extremity surgery patients receiving opioid prescriptions from multiple unique prescribers during the perioperative period. We observed that patients who had multiple unique opioid prescribers were both more likely to fill additional opioid prescriptions and demonstrate prolonged opioid use postoperatively compared to patients with only a single unique prescriber. Additionally, among patients who filled multiple opioid prescriptions postoperatively, those who had multiple unique prescribers received more prescriptions than patients who received all refills from a single prescriber. Further, while all initial postoperative opioid prescriptions were written by an orthopedic provider, within six months postoperatively, almost 3/4 of opioid prescriptions filled were written by non-orthopedic providers. The postoperative prescriptions written by non-orthopedic providers were significantly larger and of longer duration than those written by orthopedic providers, which could substantially increase the patients’ risk of developing opioid dependence [23].

To reduce the risk of patients being overprescribed opioids from multiple prescribers, states have introduced online PDMP databases for providers to review before prescribing opioids [23]. PDMP websites have been proven to reduce the national opioid burden and have shown to be accurate in prescription reporting, demonstrating a 97.1% accuracy with sensitivities and specificities of 96.4% and 97.1%, respectively [24]. This resource has proven invaluable for medical providers and has undoubtedly assisted providers in identifying patients receiving excessive opioids. Despite the utility and success of PDMP systems, postoperative patients remain susceptible to opioid overprescription and its detrimental effects.

Our findings are consistent with those of previous studies which have found that non-orthopedic providers represent the highest prescribers of opioids among medical specialties. Specifically, general practitioners, including primary care, family medicine, and internal medicine, have consistently been found to be the highest opioid prescribers [16,25]. Previous studies coupled with our current findings emphasize the importance of provider consistency in perioperative medical care, as well as the importance of utilizing resources such as PDMP databases when prescribing opioids. When orthopedic surgery patients have increased analgesic requirements postoperatively, it should be emphasized that they return to their surgeon’s practice for evaluation and further care. This not only ensures proper diagnostic workup to rule out potential postoperative complications but will also ensure that patients are not overprescribed opioids by non-orthopedic providers who may not be as experienced in perioperative patient care and analgesia requirements.

Previous studies have shown larger quantities of opioids prescribed postoperatively could negatively affect patient outcomes [26]. The present study found that non-orthopedic providers prescribed significantly larger and longer-duration opioid prescriptions postoperatively, which could place patients at risk for developing opioid dependence and a multitude of medical complications [8]. This is especially meaningful given the dose-dependent increased risk for patient mortality associated with opioids, which has been shown to increase nearly nine times when prescribed opioid doses exceeding 100 MMEs/day [27]. A longer duration of opioid prescription appears to have similar negative effects, with increased duration of opioid use being found to increase the risk of dependence in opioid-naïve patients [28].

In general, postoperative orthopedic patients have an inherent risk of developing prolonged opioid use [29]. Prior orthopedic studies have investigated risk factors contributing to prolonged postoperative opioid use, including age, sex, socioeconomic status, comorbidity scores, prescription sizes, concurrent mental health diagnoses, tobacco and drug use, and preoperative opioid use. Identifying patients at increased risk for opioid-related postoperative complications is an important clinical consideration for orthopedic surgeons and can guide postoperative patient analgesia strategies. Surgeons can minimize opioid prescription by utilizing multimodal pain regimens, which have proven equally effective for postoperative pain control as opioid-only regimens in hand and upper extremity surgery [30]. Additionally, surgeons are encouraged to employ other interventions proven to decrease opioid consumption, such as preoperative patient counseling and uniform opioid prescribing guidelines [29].

Our study has several limitations. First, the retrospective nature of the study has its innate limitations. Second, data from the PDMP database provides a record of filled opioid prescriptions but does not provide insight into opioid consumption patterns. Third, it is possible patients obtained and used opioids through means that would not be recorded in the PDMP system, such as illicitly or from old leftover prescriptions. Fourth, the PDMP offers information on controlled substance prescriptions that were dispensed but does not provide the reason for patients obtaining these prescriptions. It is entirely possible that opioid refills were written for concurrent comorbidities or new injuries treated at outside institutions. Lastly, we included three common orthopedic hand and upper extremity procedures in our analysis to increase the generalizability of our study findings. It is possible that patients’ pain and opioid prescription filling experiences may differ between the three procedures analyzed.

## Conclusions

This study revealed that patients with multiple individual opioid prescribers during the perioperative period are at higher risk for prolonged opioid use postoperatively compared to patients with a single opioid prescriber. Additionally, non-orthopedic providers were the highest prescribers of opioids postoperatively, prescribing significantly larger and longer opioid prescriptions compared to orthopedic prescribers. These findings emphasize the importance of utilizing PDMP systems when prescribing opioids to orthopedic surgery patients. Additionally, increasing single provider consistency throughout the perioperative period following hand and upper extremity surgery can help to prevent opioid overprescription and potentially adverse opioid-related outcomes.
